# FASN, SCD1 and ANXA9 gene polymorphism as genetic predictors of the fatty acid profile of sheep milk

**DOI:** 10.1038/s41598-021-03186-y

**Published:** 2021-12-09

**Authors:** Ewa Pecka-Kiełb, Inga Kowalewska-Łuczak, Ewa Czerniawska-Piątkowska, Bożena Króliczewska

**Affiliations:** 1 Department of Biostructure and Animal Physiology, Faculty of Veterinary Medicine, Wroclaw University of Environmental and Life Sciences, Norwida 31, 50-375 Wrocław, Poland; 2 Department of Genetics, Faculty of Biotechnology and Animal Husbandry, West Pomeranian University of Technology in Szczecin, Piastów Avenue 45, 79-311 Szczecin, Poland; 3 Department of Ruminant Science, Faculty of Biotechnology and Animal Husbandry, West Pomeranian University of Technology in Szczecin, Klemensa Janickiego 29, 71-270 Szczecin, Poland

**Keywords:** Biotechnology, Genetics

## Abstract

In this study, single nucleotide polymorphisms (SNPs) in the *ANXA9* (annexin 9), *FASN* (fatty acid synthase) and *SCD1* (stearoyl-CoA desaturase 1) genes were analyzed as factors influencing fatty acid profiles in milk from Zošľachtená valaška sheep. SNP in selected genes was identified using polymerase chain reaction (PCR) and restriction fragment length polymorphism (PCR–RFLP). The long-chain fatty acids profile in sheep milk was identified by gas chromatography. Statistical analysis of the *SCD1/Cfr*13I polymorphism showed that the milk of the homozygous *AA* animals was characterized by a lower (*P* < 0.05) share of C4:0, C6:0, C8:0, C10:0, C12:0, C14:0 in comparison to the homozygous *CC* sheep. The milk of heterozygous sheep was characterized by a higher (*P* < 0.05) proportion of C13:0 acid compared to the milk of sheep with the homozygous *AA* type. A higher (*P* < 0.05) level of saturated fatty acids (SFA) was found in the milk of *CC* genotype sheep compared to the *AA* genotype. Our results lead to the conclusion that the greatest changes were observed for the *SCD1/Cfr*13I polymorphism and the least significant ones for *FASN/Aci*I. Moreover, it is the first evidence that milk from sheep with *SCD1/Cfr13I* polymorphism and the homozygous *AA* genotype showed the most desirable fatty acids profile.

## Introduction

Diseases of Civilization and the rapid development of food production have resulted in new consumer food trends. Modern consumers are looking for products rich in valuable nutrients, vitamins and substances that have a positive effect on human health. In contrast, excessive consumption of the SFA highly increases coronary disease risk, diabetes, obesity, atherosclerosis, and high low-density lipoprotein (LDL) levels^1^. In food, unsaturated fatty acids have pro-health properties, in particular, they contribute to reducing blood cholesterol^2^. The most desirable fatty acids in the human diet are conjugated linoleic acid (CLA), eicosapentaenoic acid (C20:5n-3, EPA) and docosahexaenoic acid (C22:6n-3, DHA), which play an important role in preventing cancer, cardiovascular, autoimmune, and psychological diseases^3,4^.

Sheep milk is a rich source of unsaturated fatty acids compared to cow and goat milk^5^. The composition of milk, including the fatty acids profile, is determined by environmental and genetic factors^6–10^ and was valuably reviewed by Lazar et al.^11^.

The candidate gene approach is applied concerning genes whose products might affect production traits. The most extensively studied genes are genes encoding milk proteins (e.g. caseins), hormones and their receptors. Other genes of interest in this area include genes encoding enzymes that participate in fatty acid metabolism as well as genes encoding fatty acid binding and transport proteins^12^.

Fatty acid synthase (FAS) encoded by the *FASN* gene is a multifunctional homodimeric enzyme that catalyzes the synthesis of fatty acids (FA), plays a key role in the synthesis of short- and medium-chain fatty acids in mammals^13,14^. Additionally, which is important in the adult life of mammals, it determines the energy homeostasis of the organism and is involved in the production of milk lipids during lactation^15^. Annexin 9 (ANXA9) encoded by the *ANXA9* gene is a phospholipid and Ca^2+^ binding protein. It is also involved in the transport across the cytoplasmic membrane, which is important in the mammary gland^16^. On the other hand, stearoyl-CoA desaturase 1 (SCD1) encoded by the *SCD1* gene is the key and a rate-limiting enzyme involved in the metabolism of mammary lipids and is responsible for the conversion of SFA into monounsaturated fatty acids (MUFA). Also be a key enzyme in the production of the cis-9, trans-11 isomer of conjugated CLA. CLA can be found in ruminant milk and tissue fat and is considered a beneficial effect on human health^17,18^.

Dairy sheep farming systems vary from extensive to intensive according to the economic relevance of the production chain and the specific environment and breed^19^. Only a few publications have comparative values of milk composition for different breeds of sheep and milk energy of sheep breeds, but milk components were not different among breeds^19^.

The available literature data have shown the effects of SNP on the basic composition and protein fractions in sheep milk, however, each variant of the polymorphism was considered separately for each of the *FASN*, *ANXA9* and *SCD1* genotypes^8,20,21^. Therefore, an attempt was made to determine which of the studied polymorphisms has a more significant impact on the proportion of fatty acids in sheep milk and could be the best candidate marker. Additionally, Zošľachtená valaška sheep are a local Slovak breed, which opens up prospects for future selection programs and animal protection strategies. For this purpose, this study analyzed SNP polymorphisms in the *ANXA9, FASN* and *SCD1* genes as a factor influencing the fatty acid profile in the milk of Zošľachtená valaška sheep.

## Materials and methods

### Animals and nutrition

The experiment was carried out in a flock of sheep of the Zošľachtená valaška a perspective breed, mainly for mountainous areas, which is included in the native Slovak breed. They are bred in the three Slovak regions (Spiš, Orava, and Liptov). The breed was generated by the intentionally combined crossing of Native Wallachian sheep with the rams of various imported breeds as, Cheviot, Hampshire, Lincoln, Texel and Leicester. The targeted crossing breed resulted in the improvement of the qualitative and quantitative properties of wool production, live weight, milk production while maintaining good walking ability and adaptation to worse climatic conditions and was recognized as a semi-fat breed with combined performance parameters (meat, milk, wool). The breeding program of Zošľachtená valaška sheep is aimed at improving genetic indicators and is still being developed towards breed, aimed at improving the production of milk and meat, and thus creating a meat-and-dairy utility type, therefore it is genetically interesting for study genes polymorphism involved in milk production according to their function and effects. At present, 128,930 animals of this breed are kept in Slovakia^22^. Animals of this breed are characterized by good adaptation to difficult mountain conditions. Ewes weigh from 50 to 55 kg, they are seasonally polyoestrous during the fall season (October – November). Breed, age and stage of lactation have a significant impact on changes in sheep's milk. Therefore, fifty sheep were selected from a herd, taking into account age and lactation stage to eliminate the main variables influencing milk parameters. After lamb weaning, the ewes produced 80–120 kg of milk during the 150 days of lactation.

### Ethical statement

All methods and procedures strictly complied with the “Regulation on the Studying Procedures and Principles of Animal Experiments of Ethics Committees” and were approved by the Veterinary Care of the University of Veterinary Medicine and Pharmacy in Košice, permission number IČO 00,397,474 2015, licensed by the Ministry of Education, Sciences, Research and Sport of the Slovak Republic All animals used in this study were handled in strict accordance with good clinical practices following EU legislation (Council Directive 2010/63/EU), and all efforts were made to minimize suffering.

### Milk and blood sampling

The material for the study was collected from 50 ewes in the similar phase of milking (25–30 days of lactation) and lactation (1st and 2nd lactation). In the lambing period, the sheep were kept in sheepfold complying with the European Union Directive (No. 116, item 778, 2010) and were fed hay ad libitum, 250 g/ewe/day of wheat middlings, and 3 kg/ewe/day of haylage. To collect milk samples from ewes, their lambs were separated overnight. The milk was collected into sterile containers and transported to the laboratory at 4 °C. Besides, peripheral blood samples were collected from the external jugular vein (EJV) of ewes into test tubes containing anticoagulant (tripotassium ethylenediaminetetraacetic acid, K_3_EDTA) for DNA isolation and immediately transported to the laboratory, and frozen at − 20 °C for subsequent analysis.

### Genetic polymorphism analysis

DNA isolation was performed using the MasterPure DNA Purification Kit for Blood, Version II (Lucigen, Middleton, WI, USA) according to the manufacturer's instructions. Animal genotyping was performed using PCR–RFLP. Three SNPs in the *ANXA9* gene (intron 4, intron 5, GenBank: AY785286.1) and one in the *FASN* gene (exon 32, GenBank: GQ150557.1) and *SCD1* (promoter region, GenBank: FJ513370.1) were analyzed. Table 1 shows the location of individual SNPs and the appropriate primers, designed using the Primer3 software (http://bioinfo.ut.ee/primer3-0.4.0/), enabling the amplification of selected fragments of the analyzed genes. In the case of the reverse primer for the *FASN* gene, a mismatched nucleotide was introduced to create a cleavage site for the enzyme (this nucleotide is underlined in Table 1). The polymerase chain reaction (PCR) reaction was performed in a final volume of 25 μl, using 2 × PCR Master Mix (A&A Biotechnology, Gdynia, Poland),) containing 50 ng genomic DNA and 5 pmol of each primer. DNA amplification was performed using an initial denaturation at 94 °C for 5 min, followed by 30 cycles of 30 s denaturation at 94 °C each, annealing at a temperature appropriate for each gene for 30 s and extension at 72 °C for 30 s, ending with a final extension of 8 min at 72 °C (Table 1).

**Table 1:** Localization of SNPs and primer sequences for the tested genes.

Gene	Location	Primer Sequence (5′–3′)	Annealing temperature
*SCD1* Chromosome 22	Promoter region (31 C > A)	F: CAGGGGCAGGGGCAGAGGCA R: CGCTGGCAGCCGGTGACTGTG	62 °C
*FASN* Chromosome 11	Exon 32 (257 C > T)	F: TGAGATGGGGCAGCAGGCCT R: GGAACACTGTTCGCTTG**C**GGG	60 °C
*ANXA9* Chromosome 1	Intron 4 (c.172 + 181G > A) Intron 4 (c.173-27C > G) Intron 5 (c.267 + 103C > A)	F: CATTCCTGTGTGTCCGGTAC R: TCATCTCAGACCTAACCACCA	50 °C

After the amplification of selected fragments of *SCD1*, *FASN* and *ANXA9* genes, the identification of polymorphic loci in these genes was carried out using appropriately selected restriction enzymes. Individual PCR products were digested separately with restriction enzymes according to the manufacturer's recommendations. Next, the restriction fragments were separated on agarose gels with appropriately selected agarose concentrations. The restriction enzymes and the obtained restriction fragments for individual genotypes are presented in Table 2.

**Table 2:** The size of the restriction fragments for each restriction enzyme of the studied genes.

Gene	Product size (bp)	Restriction enzyme	Size of RFLP band (bp)
*SCD1*	225	*Cfr*13I	CC: 194, 31 CA: 225, 194, 31 AA: 225
*FASN*	275	*Aci*I	CC: 149, 107, 19 CT: 168, 149, 107, 19 TT: 168, 107
*ANXA9*	675	*Nla*III	GG: 252, 177, 175, 71 GA: 252, 248, 177, 175, 71 AA: 252, 248, 175
		*Hin*fI	GG: 366, 162 147, GC: 366, 227, 162 147,139 CC: 227, 162,147, 139
		*Tru*1	CC: 450, 225 CA: 450, 389, 225, 61 AA: 389, 225, 61

### Fatty acids analysis

Fats were extracted from the milk samples using the Folch method^23^. Next, the obtained fat was converted to fatty acid methyl esters using the Christopherson and Glass procedure (1969)^24^ with 2 M KOH in methanol. The fatty acid profile was determined, by using a gas chromatography method (Agilent Technologies 7890A, Agilent Technologies, Santa Clara, CA, USA), with a flame ionization detector and an HP-88 capillary column designed for the separation of fatty acid methyl esters (FAMEs) (100 m length, 25 mm i.d. × 0.20 μm). The initial oven temperature was 50 °C and was increased by 3 °C/min to 220 °C. The detector and dispenser temperatures were − 270 °C and 270 respectively.

To analyse experimental chromatograms a comparative analysis of the retention times of the fatty acid methyl ester standards (Sigma-Aldrich) was performed using the ChemStation software (Agilent Technologies, USA).

The desaturase index was also calculated of fatty acids as a ratio of unsaturated cis-9 to unsaturated + saturated cis-9 for different FAs^25,27^ as follows.

C14:1 cis-9 to C14:1 cis-9 + C14:0: desaturation index for C14:0;

C16:1 cis-9 to C16:1 cis-9 + C16:0: desaturation index for C16:0;

C18:1 cis-9 to C18:1 cis-9 + C18:0: desaturation index for C18:0;

CLA to CLA + trans 18:1: desaturation index for CLA.

### Statistical analysis

The frequencies of genotypes and alleles and the Hardy–Weinberg equilibrium for individual SNPs were calculated using the POPGENE software^27^, the effective number of alleles (Ne) was evaluated according to Kimura and Crow (1964)^28^ and expected heterozygosity (He) and the polymorphism information content (PIC) were evaluated according to Nei's methods^29^.

The statistical analysis of the influence of selected SNPs on the fatty acid profile in sheep milk was carried out in the Statistica 13.1 program (StatSoft Poland, Krakow, Poland). The results of the study were statistically analyzed using one-way ANOVA followed by a multiple comparisons Tukey Post-Hoc Test. Pearson correlation coefficient (r) with a two-tailed test of significance was conducted to examine the relationship between certain parameters.

The statistical analysis was performed using the following model:

$$\text{yij} = {\upmu} + \text{ai} + \text{eij}$$

where yij—analysed trait, µ—overall mean, ai—the effect of genotype on trait value, and eij—the effect of random error.

The correlation coefficients have been calculated with the following formula:

$${\mathrm{r}}_{\mathrm{xy}}=\frac{\sum_{\mathrm{i}=1}^{\mathrm{n}}\left({\mathrm{x}}_{\mathrm{i}}-\overline{\mathrm{x} }\right)\left({\mathrm{y}}_{\mathrm{i}}-\overline{\mathrm{y} }\right)}{\sqrt{\sum_{\mathrm{i}=1}^{\mathrm{n}}{\left({\mathrm{x}}_{\mathrm{i}}-\overline{\mathrm{x} }\right)}^{2}\sum_{\mathrm{i}=1}^{\mathrm{n}}{\left({\mathrm{y}}_{\mathrm{i}}-\overline{\mathrm{y} }\right)}^{2}}}$$

### ARRIVE guidelines

The present study has been carried out in accordance with ARRIVE guidelines.

## Results

The analysis of the obtained genotyping results suggested that for almost all polymorphic loci tested, the presence of all three possible genotypes was identified; only in the *FASN* gene, two out of three possible genotypes were identified. Table 3 shows the frequencies of genotypes and alleles and the expected heterozygosity, the effective number of alleles, PIC, and the value of χ2 calculated based on genotyping results.

**Table 3:** The frequencies of genotypes and alleles for the analyzed SNPs in the studied sheep flock.

Polymorphism	n	Genotype frequencies		Allele frequencies		He	Ne	PIC	HWE	
									χ^2^	*P* value
*SCD1*/*Cfr13*I	5 27 18	*AA* *AC* *CC*	0.10 0.54 0.36	*A* *C*	0.37 0.63	0.466	1.150	0.358	1.253	0.263
*ANXA9*/*Nla*III	23 20 7	*GG* *GA* *AA*	0.46 0.40 0.14	*G* *A*	0.66 0.34	0.449	1.814	0.348	0.591	0.442
*ANXA9*/*Hin*fI	22 12 18	*GG* *CG* *CC*	0.32 0.44 0.24	*G* *C*	0.54 0.46	0.497	1.987	0.373	14.923	0.0001
*ANXA9*/ *Tru*1	18 21 11	*CC* *CA* *AA*	0.36 0.42 0.22	*C* *A*	0.57 0.43	0.490	1.962	0.370	1.025	0.311

n – animal numbers; He – expected heterozygosity; Ne – effective number of alleles; PIC polymorphic information content, HWE Hardy–Weinberg equilibrium.

The expected heterozygosity for almost all examined loci was quite similar and ranged from 0.449 to 0.497; the only exception was the *FASN* polymorphism where two out of three genotypes were identified. In the case of the effective number of alleles, similar values were observed for most of the analyzed SNPs in the range of 1.814–1.987, except for the *SCD1* genes, where this value was 1.150. The PIC value for all tested SNPs was similar and ranged from 0.311 to 0.373, that according to the classification of Botstein et al. (1980)^30^ indicates an average polymorphism (0.25 < PIC value < 0.5). The next analyzed parameter was the Hardy–Weinberg equilibrium value (HWE). The distribution of genotypes consistent with the HWE was at *P* > 0.05. The analysis of the results collected in Table 3 shows that the *ANXA9/Hin*fI polymorphism was found to be incompatible with the Hardy–Weinberg equilibrium. We also assessed the effects of SNPs within the fatty acid synthase gene (*FASN*), however, we detected only two genotypes (*CC*, *CT*) out of 3 *FASN/Aci*I polymorphisms (without *TT*). Due to this, we did not calculate HWE for *FASN/Aci*I.

The profile of individual saturated fatty acids in sheep milk in relation to individual genotypes of the studied polymorphisms of the *SCD1* and *FASN* genes is presented in Table 4. Statistical analysis for the *SCD1/Cfr*13I polymorphism showed that the milk of individuals with the homozygous *AA* genotype was characterized by a lower (*P* < 0.05) share of butanoic acid (C4:0), hexanoic acid (C6:0), octanoic acid (C8:0), decanoic acid (C10:0), dodecane (C12:0), tetradecanoate (C14:0) than the homozygous *CC* sheep. On the other hand, the milk of sheep with the heterozygous genotype was characterized by a higher (*P* < 0.05) share of tridecanoic acid (C13:0) as compared to the milk of sheep with the *AA* genotype. A higher (*P* < 0.05) total level of saturated fatty acids (SFA) was found in the milk of the homozygous *CC* sheep compared to those of the homozygous *AA* genotype.

**Table 4:** The content of individual saturated fatty acids (SFA) in sheep for the *SCD1* and *FASN* polymorphisms.

	*SCD1/Cfr*13I			*FASN/Aci*I	
	AA	CA	CC	CC	CT
**SCFA**					
C4:0	0.53 ± 0.17^a^	0.54 ± 0.21	0.67 ± 0.28^b^	0.59 ± 0.23	0.58 ± 0.29
C6:0	0.68 ± 0.19^a^	0.75 ± 0.17	0.85 ± 0.21^b^	0.78 ± 0.19	0.77 ± 0.21
C8:0	0.81 ± 0.21^a^	0.92 ± 0.20	1.00 ± 0.20^b^	0.94 ± 0.21	0.95 ± 0.18
C10:0	2.79 ± 0.86^a^	3.25 ± 0.71	3.45 ± 0.72^b^	3.25 ± 0.76	3.46 ± 0.55
Total	4.80 ± 1.38	5.47 ± 1.11	5.96 ± 1.26	5.77 ± 1.07	5.56 ± 1.24
**LCFA**					
C12:0	1.92 ± 0.38^a^	2.15 ± 0.34	2.31 ± 0.40^b^	2.17 ± 0.40	2.29 ± 0.16
C13:0	0.03 ± 0.05^a^	0.06 ± 0.02^b^	0.05 ± 0.02	0.05 ± 0.03	0.07 ± 0.02
C14:0	7.22 ± 0.65^a^	7.74 ± 0.81	8.18 ± 0.89^b^	7.83 ± 0.91	8.01 ± 0.59
C15:0	1.10 ± 0.19	1.09 ± 0.19	1.12 ± 0.06	1.07 ± 0.16	1.16 ± 0.19
C16:0	21.44 ± 0.95	22.03 ± 1.15	21.81 ± 1.44	21.90 ± 1.29	21.89 ± 1.02
C17:0	1.09 ± 0.08	1.01 ± 0.11	1.01 ± 0.12	1.03 ± 0.11	0.98 ± 0.10
C18:0	12.61 ± 0.16	11.48 ± 1.41	12.33 ± 1.74	11.91 ± 1.58	11.83 ± 1.31
C20:0	0.31 ± 0.04	0.32 ± 0.04	0.33 ± 0.06	0.32 ± 0.05	0.34 ± 0.05
Total	45.70 ± 1.64	45.88 ± 2.13	47.08 ± 1.19	46.55 ± 1.33	46.27 ± 1.95
ƩSFA%	50.49 ± 2.85^a^	51.32 ± 2.85	53.02 ± 1.84^b^	51.80 ± 2.71	52.30 ± 2.27

*SCFA* short-chain fatty acids,* LCFA* long-chain fatty acids,* SFA* saturated fatty acids.

Mean ± SD values within the same row sharing a different superscript letter (a, b, c, etc.) are significantly different (*P* < 0.05).

Table 5 presents the share of unsaturated fatty acids in sheep milk in relation to the studied polymorphisms of the *SCD1* and *FASN* genes. The analysis of the *SCD1/Cfr*13I polymorphism revealed that the proportion of (Z)-11-eicosenoic acid (C20:1) in milk was lower (*P* < 0.05) in the homozygous *AA* sheep in relation to the heterozygous and homozygous *CC* individuals. Higher (*P* < 0.05) levels of MUFA were noted in the homozygous *AA* individuals compared to the homozygous *CC* individuals. The relationship for all cis-5,8,11,14,17-eicosapentaenoic acid (C20:5n3) was reverse: in the milk of the homozygous *AA* sheep the level of this acid was lower (*P* < 0.05) compared to individuals with the *CC* genotype. The polymorphism in the *FASN* gene will not statistically affect the fatty acid profile in sheep milk. For the remaining saturated and saturated fatty acids in milk, no statistical effect of polymorphisms for the *SCD1* gene was observed. Polymorphism in the *FASN*, *SDC* and *ANXA9* genes did not affect the desaturase index in sheep milk (Tables 5 and 7).

**Table 5:** The content of individual unsaturated fatty acids (UFA) in sheep milk for the *SCD1* and *FASN* polymorphisms.

	*SCD1/Cfr*13I			*FASN/Aci*I	
	AA	CA	CC	CC	CT
**MUFA**					
C14:1	0.53 ± 0.15	0.57 ± 0.09	0.56 ± 0.08	0.56 ± 0.09	0.62 ± 0.08
C16:1	5.63 ± 0.66	5.78 ± 0.73	5.44 ± 0.64	5.59 ± 0.69	5.93 ± 0.75
C17:1	0.53 ± 0.08	0.50 ± 0.06	0.49 ± 0.06	0.50 ± 0.06	0.48 ± 0.06
C18:1n9c	23.57 ± 1.35	22.75 ± 2.31	21.85 ± 1.67	22.66 ± 2.16	21.51 ± 0.94
C18:1n9t	1.92 ± 0.51	1.84 ± 0.37	1.88 ± 0.49	1.85 ± 0.37	1.94 ± 0.66
C18:1n7t	2.27 ± 0.19	2.15 ± 0.27	2.06 ± 0.27	2.13 ± 0.28	2.09 ± 0.23
C20:1	0.06 ± 0.04^a^	0.08 ± 0.03^b^	0.08 ± 0.02^b^	0.08 ± 0.03	0.08 ± 0.02
**Σ** MUFA	34.51 ± 2.00^b^	33.66 ± 2.29	32.36 ± 1.60^a^	32.63 ± 1.24	33.36 ± 2.24
**PUFA**					
C18:2n6c	1.91 ± 0.33	1.76 ± 0.29	1.77 ± 0.23	1.78 ± 0.27	1.74 ± 0.31
CLA	1.29 ± 0.12	1.25 ± 0.27	1.19 ± 0.15	1.24 ± 0.23	1.22 ± 0.18
C18:3n3	1.46 ± 0.08	1.53 ± 0.23	1.48 ± 0.19	1.53 ± 0.21	1.35 ± 0.10
C20:4n6	0.10 ± 0.07	0.11 ± 0.03	0.10 ± 0.02	0.10 ± 0.03	0.12 ± 0.04
C20:5n3	0.07 ± 0.05^a^	0.08 ± 0.02	0.09 ± 0.02^b^	0.08 ± 0.03	0.08 ± 0.02
**Σ** PUFA	4.82 ± 0.40	4.72 ± 0.66	4.62 ± 0.45	4.49 ± 0.44	4.73 ± 0.59
Σ UFA	40.14 ± 1.73	39.17 ± 2.61	37.74 ± 1.73	38.87 ± 2.49	37.92 ± 1.23
**Desaturase index**					
C14	0.07 ± 0.01	0.07** ± **0.01	0.06** ± **0.01	0,07** ± **0,01	0.07** ± **0.01
C16	0.21** ± **0.02	0.21** ± **0.02	0.20** ± **0.02	0,20** ± **0,02	0.21** ± **0.03
C18	0.69** ± **0.01	0.70** ± **0.03	0.68** ± **0.03	0,69** ± **0,03	0.68** ± **0.03
CLA	0.24** ± **0.03	0.24** ± **0.04	0.23** ± **0.03	0,24** ± **0,04	0.23** ± **0.04

*MUFA* monounsaturated fatty acids,* PUFA* Polyunsaturated fatty acids.

Mean ± SD values within the same row sharing a different superscript letter (a, b, c, etc.) are significantly different (*P* < 0.05).

The analysis of the share of short- and long-chain saturated fatty acids with respect to the individual genotypes of the studied polymorphisms in the *ANXA9* gene is presented in Table 6. Statistical analysis for the *ANXA9/Hin*fI polymorphism showed that the milk of individuals with the homozygous *CC* genotype was characterized by a higher (P < 0.05) content of pentadecanoic acid (C15:0) and eicosanoic acid (C20:0) than the milk of heterozygous sheep. In contrast, the milk of sheep with the *CG* genotype was characterized by a lower (*P* < 0.05) proportion of octadecanoic acid (C18:0) compared to the milk of sheep with the homozygous *GG* genotype. For the remaining saturated fatty acids in milk, no statistical effect of *ANXA9* polymorphisms was observed.

**Table 6:** The content of individual saturated fatty acids (SFA) in the milk of the Zošľachtená valaška sheep for the *ANXA9* polymorphisms.

	*ANXA9/Nla*III			*ANXA9/Hin*fI			*ANXA9/Tru1I*		
	AA	GA	GG	CC	CG	GG	AA	AC	CC
**SCFA**									
C4:0	0.46 ± 0.20	0.64 ± 0.29	0.57 ± 0.18	0.63 ± 0.27	0.53 ± 0.18	0.63 ± 0.27	0.58 ± 0.21	0.58 ± 0.25	0.63 ± 0.27
C6:0	0.68 ± 0.19	0.83 ± 0.20	0.77 ± 0.17	0.83 ± 0.21	0.76 ± 0.19	0.77 ± 0.17	0.79 ± 0.19	0.78 ± 0.20	0.77 ± 0.17
C8:0	0.82 ± 0.20	0.98 ± 0.19	0.94 ± 0.21	0.97 ± 0.21	0.95 ± 0.22	0.91 ± 0.19	0.97 ± 0.21	0.95 ± 0.22	0.87 ± 0.16
C10:0	2.87 ± 0.78	3.38 ± 0.64	3.31 ± 0.78	3.36 ± 0.70	3.36 ± 0.79	3.13 ± 0.71	3.40 ± 0.82	3.31 ± 0.73	3.00 ± 0.55
Total	4.83 ± 1.14	5.21 ± 1.11	5.59 ± 1.20	5.78 ± 1.26	5.60 ± 2.27	5.36 ± 1.11	5.26 ± 0.91	5.61 ± 1.28	5.74 ± 1.30
**LCFA**									
C12:0	2.03 ± 0.36	2.23 ± 0.30	2.20 ± 0.44	2.23 ± 0.32	2.23 ± 0.42	2.11 ± 0.36	2.23 ± 0.42	2.22 ± 0.36	2.04 ± 0.30
C13:0	0.06 ± 0.03	0.05 ± 0.02	0.05 ± 0.03	0.06 ± 0.01	0.05 ± 0.03	0.05 ± 0.03	0.05 ± 0.03	0.05 ± 0.02	0.05 ± 0.03
C14:0	7.98 ± 1.12	7.84 ± 0.80	7.84 ± 0.89	8.02 ± 0.95	7.91 ± 0.83	7.66 ± 0.86	7.83 ± 0.89	7.97 ± 0.91	7.66 ± 0.74
C15:0	1.13 ± 0.16	1.09 ± 0.18	1.06 ± 0.16	1.17 ± 0.15^b^	1.04 ± 0.17^a^	1.07 ± 0.17	1.08 ± 0.12	1.08 ± 0.19	1.11 ± 0.19
C16:0	22.59 ± 1.19	21.81 ± 1.26	21.79 ± 1.23	22.36 ± 1.42	22.07 ± 0.91	21.31 ± 1.30	21.78 ± 1.18	21.77 ± 1.39	22.42 ± 0.92
C17:0	1.03 ± 0.09	1.03 ± 0.11	1.00 ± 0.11	1.04 ± 0.10	0.98 ± 0.11	1.05 ± 0.10	1.03 ± 0.11	1.01 ± 0.12	1.02 ± 0.07
C18:0	11.74 ± 0.98	12.22 ± 1.78	11.65 ± 1.43	11.80 ± 1.78	11.29 ± 1.03^a^	12.74 ± 1.56^b^	11.52 ± 1.16	12.22 ± 1.83	11.80 ± 1.32
C20:0	0.33 ± 0.05	0.33 ± 0.05	0.31 ± 0.05	0.34 ± 0.04^b^	0.30 ± 0.04^a^	0.33 ± 0.05	0.30 ± 0.04	0.33 ± 0.06	0.33 ± 0.05
Total	46.87 ± 2.02	46.58 ± 1.71	45.88 ± 1.82	47.01 ± 2.09	45.87 ± 15.91	46.20 ± 1.61	46.41 ± 2.66	46.64 ± 1.44	45.79 ± 1.86
Σ SFA%	51.69 ± 3.28	52.39 ± 2.36	51.46 ± 2.72	52.79 ± 2.65	51.47 ± 2.86	51.72 ± 2.27	51.67 ± 3.03	52.24 ± 2.29	51.67 ± 3.04

*SCFA* short-chain fatty acids,* LCFA* long-chain fatty acids,* SFA* saturated fatty acids; (g/100 g of fat).

Mean ± SD values within the same row sharing a different superscript letter (a, b, c, etc.) are significantly different (*P* < 0.05).

Table 7. shows the contribution of UFA in Zošľachtená valaška milk with respect to individual genotypes of the *ANXA9* polymorphisms examined in this study. Analysis of the *ANXA9/Nla*III polymorphism showed that the share of (all-Z) -5,8,11,14-eicosatetraenoic acid (20:4n6) in milk was lower (*P* < 0.01) in the sheep with the homozygous *AA* genotype in relation to individuals with heterozygous and homozygous *GG* genotypes. In the case of the *ANXA9/Hin*fI polymorphism, it was shown that the milk of sheep with the homozygous *GG* genotype was characterized by a higher proportion of trans-octadecenoic acid (C18:1n9t) compared to the homozygous *CC* (*P* < 0.01) and heterozygous (*P* < 0.05) sheep. Also, a higher (*P* < 0.05) share of CLA was found in the homozygous *GG* individuals compared to heterozygous ones. For the *ANXA9/Tru*1I polymorphism, it was found that animals with the homozygous *AA* genotype were characterized by a lower (*P* < 0.05) proportion of C18:1n9t acid in milk compared to heterozygous sheep and higher (*P* < 0.05) compared to the homozygous *CC* animals. The polymorphism in the *ANXA9* gene did not statistically affect the share of other unsaturated fatty acids in sheep milk.

**Table 7:** The content of individual unsaturated fatty acids (UFA) in the milk of the Zošľachtená valaška sheep for the *ANXA9* polymorphisms.

	*ANXA9/Nla*III			ANXA9/HinfI			*ANXA9/Tru*1I		
	AA	GA	GG	CC	CG	GG	AA	CA	CC
**MUFA**									
C14:1	0.55 ± 0.07	0.58 ± 0.08	0.56 ± 0.11	0.60 ± 0.06	0.55 ± 0.09	0.57 ± 0.08	0.55 ± 0.08	0.58 ± 0.08	0.55 ± 0.11
C16:1	5.95 ± 0.78	5.57 ± 0.70	5.61 ± 0.69	5.55 ± 0.70	5.66 ± 0.72	5.68 ± 0.71	5.69 ± 0.58	5.69 ± 0.73	5.42 ± 0.85
C17:1	0.50 ± 0.02	0.51 ± 0.07	0.49 ± 0.06	0.51 ± 0.05	0.50 ± 0.06	0.49 ± 0.07	0.49 ± 0.08	0.50 ± 0.05	0.51 ± 0.05
C18:1n9c	21.87 ± 1.80	22.37 ± 1.95	22.77 ± 2.26	22.06 ± 1.75	22.62 ± 1.98	22.66 ± 2.44	22.79 ± 2.04	21.87 ± 1.64	23.40 ± 2.70
C18:1n9t	2.03 ± 0.47	1.85 ± 0.48	1.82 ± 0.35	1.76 ± 0.55^A^	1.80 ± 0.40^a^	2.02 ± 0.28^Bb^	1.83 ± 0.33^ab^	1.95 ± 0.47^a^	1.70 ± 0.41^b^
C18:1n7t	2.10 ± 0.29	2.07 ± 0.26	2.18 ± 0.28	2.04 ± 0.32	2.18 ± 0.26	2.12 ± 0.24	2.15 ± 0.26	2.09 ± 0.28	2.16 ± 0.30
C20:1	0.08 ± 0.07	0.07 ± 0.01	0.08 ± 0.03	0.08 ± 0.02	0.08 ± 0.04	0.07 ± 0.03	0.08 ± 0.04	0.08 ± 0.02	0.07 ± 0.01
Σ MUFA	33.06 ± 1.92	33.01 ± 1.93	33.50 ± 2.26	32.57 ± 1.88	28.90 ± 11.63	33.82 ± 2.16	32.75 ± 1.66	32.75 ± 1.66	33.57 ± 4.73
**PUFA**									
C18:2n6c	1.66 ± 0.20	1.78 ± 0.27	1.81 ± 0.29	1.79 ± 0.27	1.79 ± 0.27	1.75 ± 0.25	1.77 ± 0.25	1.73 ± 0.27	1.88 ± 0.33
CLA	1.16 ± 0.28	1.18 ± 0.23	1.30 ± 0.19	1.21 ± 0.24	1.0 ± 0.24^b^	1.16 ± 0.17^a^	1.26 ± 0.24	1.20 ± 0.18	1.24 ± 0.30
C18:3n3	1.45 ± 0.14	1.54 ± 0.26	1.48 ± 0.16	1.53 ± 0.24	1.46 ± 0.16	1.53 ± 0.23	1.53 ± 0.22	1.47 ± 0.19	1.52 ± 0.23
C20:4n6	0.07 ± 0.04^A^	0.11 ± 0.03^B^	0.11 ± 0.03^B^	0.10 ± 0.03	0.11 ± 0.03	0.09 ± 0.04	0.10 ± 0.02	0.10 ± 0.02	0.11 ± 0.04
C20:5n3	0.08 ± 0.04	0.09 ± 0.03	0.08 ± 0.02	0.09 ± 0.02	0.08 ± 0.02	0.08 ± 0.03	0.08 ± 0.03	0.09 ± 0.02	0.08 ± 0.02
Σ PUFA	4.42 ± 0.41	4.69 ± 0.59	4.76 ± 0.5	4.71 ± 0.66	4.14 ± 1.72	4.56 ± 0.51	4.73 ± 0.52	4.59 ± 0.52	2.47 ± 0.53
Σ UFA	38.30 ± 2.51	38.47 ± 2.12	39.08 ± 2.58	38.03 ± 2.20	38.95 ± 2.56	39.00 ± 2.25	39.13 ± 2.56	38.15 ± 1.95	39.36 ± 2.80
**Desaturase index**									
C14	0.06 ± 0.01	0.07 ± 0.01	0.07 ± 0.01	0.07 ± 0.01	0.06 ± 0.01	0.07 ± 0.01	0.07 ± 0.01	0.07 ± 0.01	0.07 ± 001
C16	0.21 ± 0.03	0.20 ± 0.02	0.20 ± 0.02	0.20 ± 0.02	0.20 ± 0.02	0.21 ± 0.02	0.19 ± 0.03	0.21 ± 0.02	0.21 ± 0.02
C18	0.69 ± 0.02	0.68 ± 0.04	0.70 ± 0.03	0.69 ± 0.04	0.70 ± 0.03	0.68 ± 0.03	0.70 ± 0.04	0.68 ± 0.03	0.70 ± 0.03
CLA	0.22 ± 0.04	0.23 ± 0.04	0.25 ± 0.03	0.24 ± 0.04	0.25 ± 0.04	0.22 ± 0.03	0.24 ± 0.05	0.23 ± 0.03	0.24 ± 0.04

*MUFA* monounsaturated fatty acids,* PUFA* Polyunsaturated fatty acids; (g/100 g of fat).

Mean ± SD values within the same row sharing a different superscript letter (a, b, c, etc.) are significantly different (P < 0.05) and the superscript capital letter (A, B, C, etc.) different at *P *< 0.01.

Furthermore, we estimated the genetic correlations among individual FAs, which were shown in (Tables 8 and 9). For two individual SCFA (C4:0, C6:0), and one LCFA (C:14) negative genetic correlations were observed for SCD1/Cfr13I polymorphism and two negative correlations for two LCFA (C15:0, C20:0) over ANXA9/HinfI polymorphism.

**Table 8:** Correlation coefficients between the polymorphism in genes and the content of individual saturated fatty acids (SFA) in sheep of milk.

	*SCD1/Cfr*13I	*FASN/Aci*I	ANXA9/NlaIII	ANXA9/HinfI	ANXA9/Tru1I
**SCFA**					
C4:0	− 0.3734*	0.0591	0.2211	− 0.1925	0.0956
C6:0	− 0.3742*	0.0375	0.2350	− 0.1748	0.0042
C8:0	− 0.2999	0.0636	0.2420	− 0.0920	− 0.0972
C10:0	− 0.2405	0.1467	0.2453	− 0.0467	− 0.1456
Total	− 0.3192*	0.1145	0.2630	− 0.1054	− 0.0846
**LCFA**					
C12:0	− 0.3043	0.1499	0.1894	− 0.0476	− 0.1140
C13:0	− 0.0231	0.2420	0.0153	− 0.1343	− 0.0352
C14:0	− 0.3670*	0.1308	− 0.0542	− 0.1159	0.0153
C15:0	0.0536	0.2259	− 0.0184	− 0.3889*	0.1071
C16:0	− 0.0012	0.0553	− 0.1496	− 0.1792	0.2070
C17:0	− 0.0406	− 0.1041	0.0068	− 0.2491	0.0516
C18:0	− 0.1226	− 0.0989	0.1022	− 0.0513	0.0874
C20:0	− 0.0942	0.0682	0.1736	− 0.3627*	0.1886
Total	− 0.3288*	0.0635	0.0008	− 0.2801	0.2082
ƩSFA%	− 0.3808*	0.0975	0.1210	− 0.2490	0.1096

*SCFA* short-chain fatty acids,* LCFA* long-chain fatty acids,* SFA* saturated fatty acids; (g/100 g of fat).

*Significance at* P* < 0.05 was marked by an asterisk.

**Table 9:** Correlation coefficients between the polymorphism in genes and the content of individual unsaturated fatty acids (UFA) in the milk of sheep.

	*SCD1/Cfr*13I	*FASN/Aci*I	ANXA9/NlaIII	ANXA9/HinfI	ANXA9/Tru1I
**MUFA**					
C14:1	− 0.0424	0.3085	0.1773	− 0.2766	0.1236
C16:1	0.2792	0.0831	− 0.1110	0.1654	− 0.2709
C17:1	0.0187	− 0.0681	0.0297	− 0.0887	0.1980
C18:1n9c	0.2592	− 0.1532	− 0.0140	0.0911	0.0871
C18:1n9t	0.1201	− 0.1344	− 0.1151	0.2373	− 0.2861
C18:1n7t	0.2037	− 0.0119	− 0.1108	0.2133	0.0356
C20:1	− 0.1999	0.0528	− 0.0688	− 0.0076	− 0.0863
**Σ** MUFA	0.3576*	− 0.1208	− 0.0717	0.1832	− 0.0383
**PUFA**					
C18:2n6c	0.0596	− 0.0714	0.1185	− 0.0048	0.2117
CLA	0.1565	0.0415	− 0.1189	0.1366	0.0708
C18:3n3	− 0.0284	− 0.2810	0.1501	− 0.1931	0.1059
C20:4n6	0.0232	0.1968	0.3630*	0.1773	0.0994
C20:5n3	− 0.3164*	− 0.1220	0.1814	− 0.2666	0.1570
**Σ** PUFA	0.0654	− 0.1122	0.0952	− 0.0205	0.1804
**Σ UFA**	0.3460*	− 0.1348	− 0.0451	0.1727	− 0.0089
**Desaturase index**					
C14	− 0.1992	0.2462	0.2953	0.2149	0.1108
C16	− 0.1787	0.1026	− 0.0955	− 0.0551	− 0.0097
C18	− 0.2240	− 0.0752	− 0.1707	− 0.2658	− 0.2314
CLA	− 0.0549	0.0114	− 0.0060	− 0.1211	− 0.1170

*MUFA *monounsaturated fatty acids, *PUFA *Polyunsaturated fatty acids; (g/100 g of fat).

*Significance at* P* < 0.05 was marked by an asterisk.

Positive genetic correlations were observed between MUFA and UFA and SCD1/Cfr13I, although negative correlations for C20:5n3 was observed (Table 9). We also showed a positive correlation between C20:4n6 and ANXA9/NlaIII.

## Discussion

Milk fat and fatty acid (FA) profiles affect milk and cheese quality. Some previous studies^13,31,32^, shown some genes putatively affecting the milk fat and FA content included *FASN* and the *SCD1* genes. FAS catalyzes the de novo synthesis of small- to medium-chain FAs (Crisà et al., 2010), while the SCD1 gene affects the milk FA profile and is involved in the synthesis of monounsaturated FA by introducing a double bond in the delta-9 position of C14:0, C16:0 and C18:0^13,32^ . However, the role of ANXA9 in lactation is unknown^33^.

Sheep milk is characterized by high variability in the level of fatty acids^34–36^. The level of saturated fatty acids (SFA) ranges from 49.43 to 82.9 g/100 g fat. On the other hand, the share of MUFA and polyunsaturated fatty acids (PUFA) is slightly lower (MUFA: 11.95–45.26 g/100 g fat, PUFA 2.79–12.24 g/100 g fat)^35^. The results obtained in the author's research correspond with the literature data.

Because the biohydrogenation process takes place in the rumen content, nutrition is the main factor determining the fatty acid profile in the milk of ruminants^14^. However, the level of fatty acids in sheep milk shows a low to moderate genetic variation for individual acids (ranging from 0.01 to 0.47)^35^. Therefore, nutrition and genetics play a key role in modulating the composition of milk fat^34^. Moreover, due to the crucial role of the *FASN* and *SCD1* genes in lipid metabolism and/or their location in chromosomal regions associated with milk content and quality, SNP polymorphisms within these genes allow partially explain the variation of FA composition in sheep milk.

In the case of polymorphism in the *SCD1* and *ANXA9* genes, the analyzed SNPs are mapped in the non-coding parts, i.e. the promoter region and introns, respectively. Mutations in regulatory regions such as promoters or enhancers can disrupt or create new transcription factor binding sites and cause changes in transcription regulation. Mutations in untranslated regions may affect the regulation of translation or modify the microRNA binding sites and thus affect mRNA stability^37^. The polymorphism analyzed in the *FASN* gene is an exon mapped change and it is a synonymous mutation. In our work, it was not investigated whether the analyzed mutations in the *FASN* gene directly influenced the expression of this gene. In contrast, it is generally believed that the amino acid sequence of proteins determines the expression, folding, and function of a protein, while mutations that alter the basic structure of a protein may affect these properties^38^. In general, silent mutations can modify all phases of the gene expression process, resulting in the amplification or reduction of proteins concentration. Therefore, while most silent mutations do not alter protein functionality, they can dramatically alter protein abundance^37^. In many cases, the effect of the SNP polymorphism rather regulate gene expression than changing the amino acid sequence as was shown by Knutsen et al. in bovine^39^.

Intronic mutations in the *ANXA9* gene are not located at sites involved in splicing and are not conserved. In the case of polymorphism in the promoter region of the *SCD1* gene, Garciá-Fernández et al. (2009)^40^, based on the characteristics of the promoter of the bovine *SCD1* gene, report that the 31 C > A polymorphism is located between two conserved regions that include the critical binding sites of the transcription factor. Importantly, the second conserved promoter region is the critical region for the expression of the SCD—transcriptional enhancer element (STE). This region, which is 109 bp downstream of the polymorphism understudy, contains the sterol response element-binding protein (SREBP) and PUFA response element and plays a key role in the inhibitory effect of CLA and oleic acid on SCD transcription^40^.

Stearic-CoA desaturase, also known as Δ9-desaturase, is an enzyme [EC 1.14.19.1] associated with an endoplasmic reticulum (ER) that catalyzes the formation of monounsaturated fatty acids (MUFAs) from de novo-synthesized or food-supplied saturated fatty acids (SFAs)^41^. It is a rate-limiting enzyme in monounsaturated fatty acid (MUFA) biosynthesis that introduces a cis double bond between carbons 9 and 10 in the saturated fatty acid spectrum, with a preference for C16: 0 and C18: 0. This enzyme plays a key role in lipid metabolism and the maintenance of membrane fluidity, based on the physiological importance of the ratio between saturated and monounsaturated fatty acids^40^. An extremely important function of stearyl-CoA desaturase seems to be shaping the composition of fats contained in adipose tissue and the profile of fatty acids in meat and milk of farm animals^42^. Barber et al. examined the level of SCD mRNA expression in seven types of sheep adipose tissue to demonstrate its effect on the size of fat cells, as well as the ratio of stearic acid content (C18: 0) to oleic acid content (C18: 1)^43^.

According to Crisà et al. (2010)^13^, 257*C*** > ***T* (exon 32) polymorphism in the *FASN* gene significantly affects the level of the following acids in sheep milk: C10:0; C10:1, C12:0; C14:0; C15:0; C17:1. On the other hand, other authors found the effect of the *FASN* gene polymorphism (SNP was mapped in intron 31) also on the C13:0 level in sheep milk^44^. However, it is the T allele in *FASN* that is responsible, at least in part, for a higher level of fatty acids in milk^13^^.^ In our research, the *FASN* polymorphism affected no changes in the fatty acid composition in milk, which may be related to the low share of the T allele in the sheep genotype. The study performed on sheep by Sztankoova et al. also confirmed that the SNP g.257*C* > *T (FASN)* contributes to influencing the medium- and long-chain FAs (C5:0 and C15:0), and a tendency was also observed for association with C18:1n9c and C18:3n6^45^.

Studies by other authors have shown a link between polymorphisms (other than those studied in the present work) of the *SCD1* gene and the composition of fatty acids in ruminant products, including sheep milk. They found that the analyzed SNPs in *SCD1* significantly influenced the level of C16:1 acid, C18:1 trans-11 acid, the content of SFA and MUFA in sheep milk^46^. Moreover, Gu et al. (2019)^47^ analyzed the polymorphism in the promoter region of the *SCD1* gene (g.133A > C) showed that the presence of the C allele results in higher levels of MUFA and lower levels of SFA. In our study we found, that the milk of the homozygous CC sheep contains a higher level of C20:1 and C20:5n3 fatty acids than the milk of sheep with AA genotype.

In the case of SFA and MUFA, a higher share of MUFA and a lower share of SFA were obtained in the milk of the homozygous *AA* sheep. The presence of features linked to polymorphism is related, among others, to the specificity of population and animal species^48^, which may explain the obtained results.

There are only a few studies in the available literature describing the effect of *SCD1*^46^ and *ANXA9* polymorphisms on the composition of sheep milk, including the fatty acid profile. The polymorphism in the *ANXA9* gene affects the yield of milk fat in cows^33^ and the level of fat in sheep milk^8^. Our research revealed the effect of SNP in the *ANXA9* gene on the share of the following acids: C15:0; C18:0; C20:0 and C18:19t; C20:4n6; and CLA.

Furthermore, Carta et al. (2008)^49^ performed a genome-wide scan for loci associated with FA composition in sheep's milk, where the most significant QTL that affects the fatty acid composition (chromosome-wise thresholds) associated with *FASN* were detected on OAR11 (C14:0 and C16:0) and OAR6 (MUFA). Our analysis of QTL located next to the studied genes by using Sheep QTLdb (https://www.animalgenome.org/cgi-bin/QTLdb/index) showed additional QTL on OAR11 (cis-10-C17:1; QTL 14,223; and C10:0; QTL 14,220)^50^. Analysis performed for SCD1 showed QTL on OAR22 that affect C4:0 (QTL:13,868), C16:1 (QTL:13,869;), C18:1 (QTL:13,870), C18:2(n-6) (QTL:13,871), and MUFA (QTL:13,877) content in milk. The QTL related to the ANXA9 gene and FA composition in milk has not been demonstrated in sheep. However, Calvo et al. suggested that *ANXA9* may be a candidate gene for milk production traits, as well as for SCC in cattle, concerning its localisation in the proximity of QTL for these traits^16^. This was also confirmed by Martinez et al. (2010) in a Spanish Holstein–Friesian population^33^. Furthermore, Kulig et al. (2015) have shown that there are associations between the ANXA9 polymorphism and SCC in the milk of Jersey cows^51^.

In the human diet, foods with a low n-6/n-3 share, ranging from 1:1 to 4:1, are desirable^52^. Maintaining a sufficiently low n-6/n-3 ratio in the diet has a positive effect on the cognitive functions of the body and reduces the risk of depression^53^. Additionally, the high consumption rate of (n-3)/(n-6) PUFAs reduces the risk of neoplastic diseases and inflammations^9,52,54^.

In the case of the *SCD1/Cfr*13I polymorphism, an increase in C20:5n3 was observed in milk of the sheep with homozygous *CC* genotypes, without changes in the level of n-6 acids, which may cause an increase in the value of (n-3)/(n-6). In the case of *ANXA9/Nla*III polymorphism analysis for *AA* homozygous animals, a decrease in the C20:4n6 acid level was found, which can also be considered a favourable phenomenon. Supplementation of CLA in the diet of animals and humans improves the metabolism of glucose and lipids in the body^55^. In our research, an increase in the level of CLA in the milk of sheep with the *GG ANXA9/Hin*fI genotype was observed.

According to studies by other authors, long-chain unsaturated fatty acids are important in the human diet^52,54,55^. Replacing SFA with PUFA in the human diet is beneficial for the cardiovascular system^56^. In the diet, saturated acids, such as C18:0, C14:0 and C12:0, adversely affect the increase of plasma lipoproteins^57^. On the other hand, the consumption of saturated fatty acids with a carbon chain from C12 to C16 increases the level of plasma LDL-C^56,58^. In our research, an increase in the level of C18:0 acid was noted in the *ANXA9/Hin*fI polymorphism in the homozygous GG individuals, while in the homozygous AA sheep, in the case of the *SCD1/Cfr*13I polymorphism, a decrease in the level of C12:0, C13:0, C14:0 acids and an increase in MUFA level in milk was observed. Consuming MUFA has a positive effect on humans, reduces total cholesterol and LDL fraction in the blood^59^^.^

The share of individual fatty acids in the human diet determines both physical and mental health. The present study determined the relationship between the studied polymorphisms and the fatty acid profile in milk and the results may be used in the selection program in sheep flocks: the choice of an appropriate genotype variant will ensure the desired fatty acid profile in milk.

## Conclusions

The research was aimed at searching for the best genetic marker influencing the fatty acid profile in sheep milk. These preliminary findings show that the greatest changes were observed in sheep with *SCD1/Cfr*13I polymorphism and the least significant ones in *FASN/Aci*I. Milk obtained from the homozygous *AA* sheep (*SCD1/Cfr*13I) had the best fatty acid profile. Slight changes in the milk of sheep were found for polymorphisms in the *ANXA9* gene. For the *ANXA9/Nla*III polymorphism (*AA* genotype animals) a favourable reduction in the level of C20:4n6 acid in the milk was noted. Changes were also found for the *ANXA9/Hin*fI polymorphism (homozygous *GG* individuals). The milk of these sheep was characterized by an increase in the level of C18:1n9t and CLA acids, which is beneficial; unfortunately, an undesirable increase in the proportion of C18:0 acid was also found. Because presented results indicate the association of the analyzed genotypes with the fatty acid profile in Zošľachtená valaška, a larger prospective study will be continued to explore the effect of SNPs, also in the other genes showing effects on sheep milk. Moreover, results of our study can be useful for breeders, especially that Zošľachtená valaška sheep are included in the breeding program and sheep with homozygous *AA* (*SCD1*/*Cfr*13I) could be used during the breed improvement program for genetic selection if no detrimental trait is associated with this genotype.
